# Rituximab therapy for childhood onset idiopathic nephrotic syndrome: experience of a Portuguese tertiary center

**DOI:** 10.1590/2175-8239-JBN-2022-0056en

**Published:** 2022-10-17

**Authors:** Rita Gomes, Sara Mosca, Mariana Bastos-Gomes, Liane Correia-Costa, Liliana Rocha, Ana Teixeira, Teresa Costa, Maria Sameiro-Faria, Paula Matos, Conceição Mota

**Affiliations:** 1 Centro Hospitalar Universitário do Porto, Centro Materno-Infantil do Norte, Serviço de Pediatria, Porto, Portugal.; 2 Unidade Local de Saúde do Alto Minho, Serviço de Pediatria, EPE, Viana do Castelo, Portugal.; 3 Centro Hospitalar Universitário do Porto, Centro Materno-Infantil do Norte, Serviço de Pediatria, Unidade de Nefrologia Pediátrica, Porto, Portugal.

**Keywords:** Nephrotic syndrome, Idiopathic, Rituximab, Síndrome nefrótica, Idiopática, Rituximabe

## Abstract

**Introduction:**

Rituximab (RTX) is a therapeutic option in pediatric difficult-to-treat idiopathic nephrotic syndrome (NS). We aimed to assess the efficacy and safety of RTX use in these patients.

**Method:**

A retrospective study of all patients with idiopathic NS treated with RTX was conducted in a pediatric nephrology division of a tertiary hospital. Demographic, anthropometric, clinical and analytical data were collected prior to treatment and at 6, 12, and 24 months.

**Results:**

Sixteen patients were included (11 males), with a median (25^th^–75^th^ percentile, P25–P75) age at diagnosis of 2 (2.0–2.8) years. Fifteen were steroid-sensitive and 1 was steroid-resistant and sensitive to cyclosporine. The median age at administration of RTX was 10 (6.3–14.0) years. Throughout a median follow-up time of 2.5 (1.0–3.0) years, 6 (37.5%) patients achieved partial remission and 7 (43.8%) had no relapses and were not taking any immunosuppressants at the 24-month follow-up visit. Regarding complications, 1 patient presented persistent hypogammaglobulinemia. Compared with the 12-month period before RTX, there was a decrease in the median number of relapses at 6 and 12 months [3 (3.0–4.0) vs 0 (0–0.8) and 0.50 (0–1.0), respectively; p = 0.001] and in the daily steroids dose (mg/kg/day) at 6, 12, and 24 months [0.29 (0.15–0.67)vs [0.10 (0.07–0.13); p = 0.001], [0.12 (0.05–0.22); p = 0.005] and [0.07(0.04–0.18); p = 0.021]], respectively. There was also a reduction in the median BMI z score at 24 months [2.11 (0.45–3.70) vs. 2.93 (2.01–3.98); p = 0.049].

**Conclusion:**

Our results confirm the efficacy and safety of RTX use in pediatric idiopathic NS and highlight its’ potential cardiometabolic benefits.

## Resumo

**Introdução:**

Rituximabe (RTX) é uma opção terapêutica na síndrome nefrótica (SN) idiopática pediátrica de difícil tratamento. Visamos avaliar eficácia e segurança do uso de RTX nestes pacientes.

**Método:**

Realizou-se estudo retrospectivo de todos os pacientes com SN idiopática tratados com RTX, em uma unidade de nefrologia pediátrica de um hospital terciário. Dados demográficos, antropométricos, clínicos e analíticos foram coletados antes do tratamento e aos 6, 12 e 24 meses.

**Resultados:**

Incluímos 16 pacientes (11 do sexo masculino), com idade mediana (percentil 25–75, P25–P75) de 2 (2,0–2,8) anos ao diagnóstico. Quinze eram sensíveis a esteroides, e 1 resistente a esteroides e sensível à ciclosporina.A idade mediana na administração do RTX foi 10 (6,3–14,0) anos. Durante um tempo mediano de acompanhamento de 2,5(1,0–3,0) anos, 6 (37,5%) pacientes alcançaram remissão parcial e 7 (43,8%) não tiveram recidivas e não estavam tomando imunossupressor no acompanhamento aos 24 meses. Quanto às complicações,1 paciente apresentou hipogamaglobulinemia persistente. Comparado ao período de12 meses anterior ao RTX, houve diminuição no número mediano de recidivas em 6 e 12 meses [3 (3,0–4,0) *vs* 0 (0–0,8) e 0,50 (0–1,0), respectivamente; p = 0,001] e na dose diária de esteroides (mg/kg/dia) aos 6, 12 e 24 meses [0,29 (0,15–0,67) *>vs* [0,10 (0,07–0,13); p = 0,001], [0,12 (0,05–0,22); p = 0,005] e [0,07 (0,04–0,18); p = 0,021], respectivamente. Houve também redução na mediana do escore z do IMC aos 24 meses [2,11 (0,45–3,70) *vs* 2,93 (2,01–3,98);p = 0,049].

**Conclusões:**

Nossos resultados confirmam a eficácia e segurança do uso de RTX em SN idiopática pediátrica, destacando seus potenciais benefícios cardiometabólicos.

## Introduction

Idiopathic nephrotic syndrome (NS) is the most common chronic glomerular disease in children, affecting up to16 per 100.000 children^1, 2, 3^. The exact pathogenesis of NS is unclear, but a combination of genetic predisposition, circulating factors, environmental/infectious triggers and other mechanisms has been hypothesized^4^. 

Idiopathic NS in childhood can be classified according to the International Study of Kidney Disease in Children (ISKDC) based on the response to steroids, which are the cornerstone of therapy. Most children respond well to initial steroid therapy and are considered to have steroid-sensitive nephrotic syndrome (SSNS). However, approximately 40–50% develop a complicated course resulting in frequent relapses (FRNS) or steroid dependency (SDNS)^4,5^. Moreover, 10–20% of patients do not respond to steroid therapy and are considered to have steroid-resistant nephrotic syndrome (SRNS)^3,6^. 

The prognostic factors associated with the course of NS, include the initial response to steroid therapy, the genetic profile, the response to other immunosuppressive drugs, and the duration of follow-up^6^. Kidney pathology is also a very important diagnostic and prognostic tool. SSNS is usually characterized by minimal change disease (MCD), whereas in SRNS, focal segmental glomerulosclerosis (FSGS) is the most prevalent finding^1,7^. Other frequent histologic patterns are mesangial proliferative glomerulonephritis, immunoglobulin A nephropathy or membranoproliferative glomerulonephritis. 

The management of patients with FRNS, SDNS, and SRNS is complex, involving different immunosuppressive drug schemes. Furthermore, the occurrence of adverse effects of chronic steroid therapy frequently leads to the introduction of additional second-line immunosuppressive agents, including calcineurin inhibitors (cyclosporine, tacrolimus), mycophenolate mofetil, cyclophosphamide, and levamisole^5^. However, there is no consensus regarding the choice, duration, or sequence of these drugs, as none are free of adverse effects (e.g. nephrotoxicity, neurotoxicity, hematologic side effects, and diabetogenic potential) and all require regular monitoring^1,2,5,8^. 

In fact, managing these patients is often challenging since some children continue to relapse frequently and require steroids despite multiple adjunctive therapies^4^. Collectively, at least 20% of children have complicated FRNS/ SDNS, defined by frequent relapses or steroid dependence during or after immunosuppressive therapies. Additionally, 1–3% has refractory SRNS, as they present resistance to steroids and immunosuppressive agents, putting them at high risk of evolution to kidney failure. For the aforementioned reasons, new drugs are needed to address these problems. 

In this context, Rituximab (RTX), a monoclonal anti-CD20 antibody, has become a promising treatment option in difficult-to-treat NS since its use was first described in 2012. The exact mechanism of action of RTX is still unclear but it was suggested that both immunological mechanisms and a direct effect on podocytes play a role^1^. Currently, RTX is being used in multiple centers for FRNS and SDNS, particularly when durable full remission is not achieved with other immunosuppressive drugs^3,6,7^. Its efficacy for refractory SRNS remains uncertain due to limited data^9^. RTX-induced depletion of CD19 B cells is taken as a sign of effective treatment and usually lasts for 6–8 months but therapeutic response may persist for several months, even after repopulation of B cells^6^.

With regards to safety, RTX is usually well tolerated in most children and has a limited number of adverse effects, the most common being infusion reactions^8^. Other side effects include bone marrow suppression, hypogammaglobulinemia, and an increased risk of infections^10^. Rare but potentially serious adverse effects include hepatitis B virus reactivation, RTX-associated lung injury, anaphylactic reaction, colitis, fulminant myocarditis, *Pneumocystis Jiroveci* pneumonia, or multifocal leukoencephalopathy^11^. 

RTX has been considered an efficient treatment option for children with difficult-to-treat idiopathic NS, with several previous studies showing a significantly decreased number of relapses after RTX, enabling tapering off steroids and/or other immunosuppressive agents^1,4,8,12^. Considering its good tolerability and lack of nephrotoxic effects, some authors advocate that RTX may be considered as a first-line steroids-sparing therapy in children with SDNS^5^. 

The optimal RTX regimen remains unknown^13^. In fact, data directly comparing dosing regimens are scarce and there is a significant variation in prescriptions worldwide, ranging from 375 to 1500 mg/m^2^ per treatment course^14,15^. Whilst previous reports have described a longer remission period in patients receiving high-dose RTX, recent trials have shown satisfactory outcomes with lower (375 mg/m^2^) initial doses^16,17,18,19^. Recently, an international multicenter study of 511 children conducted at 11 tertiary pediatric nephrology centers showed that the relapse-free survival in low-dose RTX was similar to medium and high doses, but only with maintenance immunosuppression, suggesting that both RTX dose and maintenance immunosuppression have important effects on the outcomes^13^. The more recent 2021 KDIGO guidelines recommend a RTX dose of 372 mg/m^2^ in a 1 to 4 infusion scheme^20^. A review from 2022 states that it appears reasonable to treat patients with severe SSNS with at least two 375 mg/m^2^ doses of RTX when complete discontinuation of other treatments is planned, and with a single 375 mg/m^2^ infusion when prophylactic therapy with another immunosuppressive agent is prescribed^11^. However, further investigation is required to establish the minimum effective dose, frequency of redosing, potential long-term toxicity, and the role of prophylactic dosing and concomitant immunosuppression. 

In the present study, we describe the experience of our center with the use of RTX in pediatric patients with difficult-to-treat idiopathic NS. We aimed to assess RTX efficacy in reducing the incidence of relapses and the need for steroids and other immunosuppressive medications over 6, 12 and 24-months follow-up by comparing to data recorded 12 months before RTX administration. We also aimed to evaluate the safety and tolerability of RTX treatment during the follow-up period. 

## Methods

### Study Design and Sample

We conducted an observational retrospective study with analysis of the clinical records of all pediatric patients aged less than 18 years with idiopathic NS involving native kidneys treated with RTX between 2017 and 2020 in our tertiary center at Centro Materno-Infantil do Norte, Centro Hospitalar Universitário do Porto, Portugal. This study was a continuation of a previous work performed in our center in 2019^21^. It should be noted that we used a larger sample and, in particular, a longer follow-up time. Patients with an identified genetic cause for NS were excluded from the analysis.

### Variable Definition and Data Collection

Regarding NS classification, patients unresponsive to prednisone for a minimum period of 8 weeks fulfilled the criteria for SRNS. FRNS was defined as ≥ 2 relapses within 6 months after initial remission or > 4 relapses within 12 months. SDNS was defined as ≥ 2 relapses during the reduction of steroid treatment or within 2 weeks after steroid discontinuation. Nephrotic syndrome was considered difficult-to-treat in steroid-resistant or steroid-dependent cases that failed to respond to at least two other immunosuppressants. Total and partial remission were defined as protein/ creatinine ratio <0.2 (mg/mg) and 0.2–2 (mg/mg) for three consecutive early-morning samples, respectively. Relapses were considered sporadic if they were < 2 in 6 months.

Data was collected from the electronic clinical files at 12 months before RTX treatment and at 6, 12, and 24 months after RTX treatment, during follow-up. Demographic (age at presentation and at RTX initiation, sex), anthropometric (weight and height), and analytical variables (serum creatinine, immunoglobulin G (IgG) levels and B cell counts; urinary proteins and creatinine) were collected. Data on NS characterization (natural history of disease, hospitalization rate, kidney histology, number of relapses) and management (previous immunosuppressive strategy including steroid dose, number of RTX infusions, and immediate and late adverse outcomes) was also collected. Height and weight were used for body mass index (BMI) calculation. Height- and BMI-for-age values were classified according to the World Health Organization (WHO) growth reference data into z-score categories. Z-score BMI values were used to classify patients in the following categories: non-overweight (−2 SD to +1 SD)and overweight/obese (> +1 SD). 

RTX was used in patients with SSNS who continued to have frequent relapses despite optimal combinations of steroids and glucocorticoid-sparing oral agents, and/or who presented serious adverse effects of therapy. Initially, our center protocol for RTX infusion included four weekly intravenous doses of 375 mg/m^2^.From 2019, we started using only two initial doses of RTX plus an on-demand approach. All doses were administered in a remission period. We use three different brands of RTX (Mabthera^®^, Ruxience^®^ and Rixathon^®^) but the hospital pharmacy ensures that the same brand is always used for subsequent administrations in the same individual patient. Pretreatment with hydroxyzine, methylprednisolone, and acetaminophen was used to prevent infusion reactions. The response to RTX was monitored by periodic clinical and analytical check-ups. Blood markers and B cell counts were evaluated on day 7 to 10 after the first RTX administration and then at every medical visit after four months, until normalization of B cell counts. Peripheral blood B cell depletion was defined as 0 cells/uL. Serum immunoglobulins were measured between day 7 and 10, at two months after the first administration, and then at every medical visit after four months, until normalization of B cells counts. Criteria for immunoglobulin G (IgG) replacement included severe hypogammaglobulinemia (< 400 mg/dl),particularly in patients experiencing recurrent infections. Urinary proteins and creatinine and serum creatinine were measured at 6, 12, and 24 months of follow-up. Serum creatinine was analyzed using a calibrator for automated system (Roche Diagnostics). To estimate glomerular filtration rate (eGFR), in mL/min/1.73m^2^, the revised Schwartz formula was used^22^.

### Ethics

This research complied with all the relevant national regulations and institutional policies and is in accordance to the tenets of the Helsinki Declaration. The study was approved by the Ethics Committee of Centro Hospitalar Universitário do Porto and Institute of Biomedical Sciences Abel Salazar. Parental consent was obtained for all patients included in the study, concerning the collection of information and biological samples.

### Statistical Analysis

Statistical analysis was performed using IBM^®^ SPSS^®^ Statistics 26.0. Continuous variables are expressed as median and 25 and 75 percentiles. Differences between groups in independent continuous variables were evaluated with Mann-Whitney test and in paired variables with Wilcoxon test. Differences in the distribution of categorical variables were evaluated with Chi-square tests. All p values were two-sided and considered statistically significant if < 0.05.

## Results

A total of 16 patients were included, 11 (68.8%) males and 5 (31.3%) females. The median age at diagnosis was 2 years (P25–P75: 2.0–2.8, minimum 1 and maximum 9). The sample baseline characterization is described in Table 1.

**Table 1.** Sample baseline characterization

**Total**	16
**Sex**	
**Male**	11 (68.8%)
**Female**	5 (31.3%)
**Classification**	
SSNS (SDNS and/ or FRNS)	15 (93.8%)
SRNS and cyclosporin sensitive	1 (6.3%)
**Median age of INS onset**	2 (2.00–2.75)
**Median age at RTX start**	10 (6.3–14.0)
**Medium duration of kidney disease before RTX**	7.54 (4.3–9.1)
**Kidney histology**	
**MCD**	10 (62.5%)
**FSGS**	3 (18.8%)
**IgM nephropathy**	3 (18.8%)
**Relapses**	3.0 (3.0–4.0)
**Anthropometric Measurements**	
z-score height	–0.55 (–1.44–0.01)
BMI (kg/m^2^)	24.85 (22.13–30.31)
z-score BMI	2.93 (2.01–3.98)
Overweight/Obesity	15 (93.8%)
**Serum creatinine**(mg/dL)	0.44 (0.34–0.51)
**eGFR**(mL/min/1.73m^2^)	185 (170–220)
**Steroid dose**(mg/kg/day)	0.29 (0.15–0.67)
**Follow-up time after RTX**(years)	2.5 (1.0–3.0)

Data is presented as number (percentage) or median (P25−P75), as appropriate. FRNS: Frequently relapsing nephrotic syndrome; SDNS: steroid-dependent nephrotic syndrome; SRNS: steroid-resistant nephrotic syndrome; MCD: minimal change disease; FSGS: focal segmental glomerulosclerosis; MPG: mesangial proliferative glomerulonephritis; BMI: body mass index; CS: corticosteroids; eGFR: estimated glomerular filtration rate; RTX: rituximab.

Regarding idiopathic NS classification, 15 (93.8%) patients presented SSNS (SDNS and/or FRNS) and1 (6.3%) SRNS, but was responsive to cyclosporine. Kidney biopsy was performed in all patients. Kidney histology found MCD in the majority of the cases(n = 10), 60% of which with immunoglobulin M deposits, FSGS in 3 cases and immunoglobulin M nephropathy in 3 patients.

Before RTX initiation, several immunosuppressants were used in different association schemes (cyclophosphamide (n = 13), cyclosporine (n = 13), mycophenolate mofetil (n = 13), levamisole (n = 1), and tacrolimus (n = 1)).

The median duration of kidney disease before RTX therapy was 7.5 years (P25–P75: 4.3–9.1), with a minimum of 11 months and maximum of 14 years and 9 months of evolution. The median age at RTX administration was 10 (P25–P75: 6.3–14.0; minimum 3 and maximum 17) years. All patients were treated with a weekly dose of 375mg/m^2^, either in 2− (n = 7; 43.8%) or 4− (n = 9; 56.3%) week schemes. Depletion of CD19 cells was confirmed after the first dose in all patients. Median serum IgG levels before RTX and at 12 and 24 months of follow-up were 401 mg/dl (P25−P75: 292.7–763.7), 59.5 mg/dl (P25–P75: 30.8–674.7) and 342 mg/dl (P25–P75: 227–670), respectively. No immediate complications were reported, but 1 patient presented persistent hypogammaglobulinemia during follow-up and was treated with mensal intravenous immunoglobulin.

Considering the 12-month period before RTX administration, the total number of relapses was 53. The median number of relapses was significantly lower before than after RTX treatment [0.5 (0−1) vs 3 (3–4); p = 0.001]. During a median follow-up time after RTX of 2.5 (P25–P75: 1.0−3.0) years, sporadic relapses occurred in 8 (50.0%) children after a median time of 8 (P25–P75: 3.1−13.7) months after the last RTX infusion. Seven (43.8%) patients evolved without relapses, 6 (37.5%) had partial remission, and 3 (18.8%) maintained the previous disease course (SD and FRNS). Half of the children recovered CD19 counts after a median of 14.0 (P25–P75: 9.3–21.0) months. Among these, 2 relapsed within the first 6 months (24 and 30 days after RTX).

With regards to medication, a statistically significant reduction in daily steroids dose (mg/kg/day) was evident at 6 [0.10 (0.07–0.13); p = 0.001],12 [0.12 (0.05–0.22); p = 0.005], and 24 [0.07 (0.04–0.18); p = 0.021] months compared to the steroid dose one year before RTX [0.29 (0.15–0.67)](Table 2). At 24 months of follow-up, 2 (12.5%) children still needed steroid therapy and 7 (43.8%) were kept on a combination of steroids and other immunosuppressants (either cyclosporine or mycophenolate mofetil). The 7 patients that evolved with no relapses did not need immunosuppressants at the 24-months follow-up after RTX.

**Table 2.** Comparison of the relapse incidence, anthropometric parameters, kidney function, and steroid doses prior to treatment and at 6, 12, and 24 months of follow-up

		6 months (n = 16)	12 months (n = 15)	24 months (n = 11)
**Relapses**		0 (0–0.75)	0.5 (0–1.00)	1 (0–3.00)
	p value	**0.001**	**0.001**	0.168
**Steroid dose**(mg/kg/day)		0.10 (0.07–0.13)	0.12 (0.05–0.22)	0.07 (0.04–0.18)
	p value	**0.001**	**0.005**	**0.021**
**Creatinine**(mg/dl)		0.46 (0.29–0.56)	0.48 (0.29–0.59)	0.49 (0.38–0.77)
	p value	0.950	0.181	**0.025**
**eGFR**(ml/min/1.73m^2^)		206 (162–239)	199 (147–244)	168 (149–205)
	p value	0.625	0.820	0.327
**z-score height**		–0.76 (–1.11–0.21)	–0.13 (–0.88–0.41)	–0.58 (–1.35–0.39)
	p value	0.535	0.105	0.875
**BMI**(kg/m^2^)		28.13 (20.64–30.94)	24.57 (20.67–31.53)	39.00 (16.96–31.29)
	p value	0.501	0.691	0.814
**z-score BMI**		2.67 (1.93–3.63)	2.37 (0.86–3.77)	0.11 (0.45–3.70)
	p value	0.408	0.363	**0.049**
**Overweight/Obesity**		15 (93.8%)	10 (66.7%)	8 (72.7%)
	p value	0.063	0.333	0.273

Data is presented as number (percentage) or median (P25−P75), as appropriate. BMI: body mass index; eGFR: estimated glomerular filtration rate.

The median zBMI at 24 months after RTX was significantly lower than the pretreatment value [2.11 (0.45–3.70) vs. 2.93 (2.01−3.98); p = 0.049]. There was also a decrease in the proportion of patients classified as overweight/ obese at 6, 12, and 24 months after RTX (93.8%, 66.7% and 72.7%, respectively, vs 93.8% before treatment), but the differences were not statistically significant. No significant differences were seen with regards to height-for-age z-scores (Table 2).

## Discussion

The treatment of idiopathic NS is challenging and often complicated by a refractory and relapsing disease course, with consequent risk of drug toxicity and progressive kidney failure. Our results are similar to those reported in previous randomized controlled trials that indicate that, among children with SDNS and FRNS, RTX is associated with lower relapse rates and longer remission periods in comparison with placebo/steroid and CNI therapy, allowing for a greater reduction or withdrawal of concomitant immunosuppressants^5,18,23,24^. The potential benefit of maintaining disease remission, combined with its limited toxicity, support its use in pediatric idiopathic NS.

Even though the ideal RTX scheme does not yet have a consensus, prior to 2019 our center’s protocol for RTX infusion included a weekly intravenous dose of 375 mg/m^2^ in a four-infusion scheme. However, several studies have demonstrated no significant difference in response rate in children between 1 and 2 doses vs. 3 to 4 doses^11,20,21^. In light of the more recent literature and the results from a previous work performed in our unit, we have changed our approach to a two-infusion scheme plus an on-demand approach, in which RTX is administered whenever there is a combined finding of NS relapse and B-cell count recovery. Recently, an international multicenter study showed that children with FRNS receiving repeated courses of RTX experienced longer relapse-free periods, but significant complications, including persistent hypogammaglobulinemia, infection and neutropenia, could occur^25^. Although their findings suggest that repeating RTX could be an effective and reasonably safe approach for most children with FRNS, the authors acknowledge that prospective trials are still needed to identify the optimal treatment approach^20,21^.

In our sample, there was a decrease in the number of relapses after RTX therapy and almost half of our patients displayed no relapses at 24 months. Similar benefits have been reported in several other studies that identified RTX as an effective treatment for patients with complicated SDNS and FRNS^3,5,8,12,21,25,26^. A multicenter, double-blind, randomized placebo controlled trial is currently being conducted to evaluate the efficacy and safety of RTX in early-stage uncomplicated SDNS and FRNS^27^ but further studies are still required to strengthen the evidence available on RTX use in NS.

Our results revealed that treatment with RTX decreased the use of steroids and other immunosuppressants, which is also in agreement with the literature^1,21,25,28^. All of the 7 patients that achieved remission are currently without immunosuppressive treatment. As for the remaining patients, 2 are under steroids and 7 need combination therapy with steroids and cyclosporine or mycophenolate mofetil, but in all patients the daily steroids dose was reduced, starting from 6-months after RTX.

In contrast to the significant benefit observed with RTX among SDNS and FRNS, its effectiveness in SRNS compared with standard therapy remains controversial. Despite the positive reports of cohort and registry studies that have shown that a significant number of patients achieve either partial or complete remission, the recent International Paediatric Nephrology Association (IPNA) guideline has given RTX a weak recommendation (grade 2C) for use in SRNS in view of the negative results of the only randomized control trial published to date^29,30^. The short follow-up period has been postulated to be one of the reasons for the study not showing promising results^31^. We reported the case of 1 patient with SRNS that was responsive to RTX, having no relapses during the first12 months of follow-up and 3 relapses during the following year, undergoing treatment with steroids only. Currently, compared to RTX, no alternative drug demonstrated superior add-on efficacy among CNI-resistant SRNS. Its role should be considered, as even partial remission has demonstrated a significantly better kidney prognosis, but future controlled trials are necessary^9,32^.

Previous studies on the impact of RTX therapy on anthropometric outcomes are scarce and often limited by small sample sizes and short follow-up periods^3,23,33,34^. A retrospective, multicenter review evaluated changes in post-RTX anthropometric parameters among children with SDNS and showed that the significant steroid sparing effect probably resulted in improved anthropometric and growth parameters at 2-year follow-up^34^. In our sample, the median zBMI was significantly lower at 24 months after RTX and there was a decrease in the proportion of patients classified as overweight/obese, albeit not statistically significant. Even though our results should be interpreted acknowledging limitations inherent to its retrospective nature, they are in accordance with the existing literature and highlight the potential cardiometabolic benefits of RTX.

The majority of studies on RTX use in pediatric NS have reported an acceptable safety profile, with infusion reactions being the most common side effect, although usually mild and transient^31^. Interestingly, in our study, no significant immediate side effects were reported. We can hypothesize that it can be related with the fact that the same brand of RTX was used for repeated doses in the same individual patient and that the hospital protocol includes premedication with hydroxyzine, methylprednisolone and acetaminophen in order to prevent infusion reactions. One girl with FRNS requiring high steroid doses (~1 mg/kg/day) and cyclosporine, underwent 2 RTX infusions by the age of 3,and exhibited persistent hypogammaglobulinemia. However, no infectious complications occurred, she achieved complete remission, and is currently under no immunosuppressive medication. No further side effects occurred during the study period. Nonetheless, further long-term prospective studies with a proper risk-benefit analysis are of utmost importance to strengthen the recommendations for RTX use.

We acknowledge a number of important limitations to our study. The most important of these is likely the short follow-up period and the fact that we report the experience of a single center. We showed that RTX treatment in difficult-to-treat idiopathic NS enabled a reduction in relapse rate and in daily steroids dose, which might have contributed to the decrease in the proportion of overweight/obesity. Thus, variables such as blood pressure, lipid profile, and waist circumference that were not evaluated, would have been very important to better characterize the cardiometabolic profile of these patients. Despite these limitations, to our knowledge, this is one of the first Portuguese cohort studies assessing RTX efficacy and safety in difficult-to-treat idiopathic NS^21^. Since it was carried out in a Pediatric Nephrology division of a tertiary center, it allowed for the inclusion of more patients, but a selection bias with inclusion of more severe cases might have occurred.

In conclusion, our results are in line with previous studies that support the efficacy and safety of RTX in SDNS, thus we believe our study reinforces RTX use as a valid therapeutic option in pediatric cases with difficult clinical management. The inclusion of RTX in immunosuppressive schemes for difficult-to-treat NS may have an impact on the quality of life of these patients and their families, potentially lessening the secondary side effects of chronic steroids and other immunosuppressants. Additionally, it might lead to a favorable economic impact due to a reduction in the number of medical visits and drug prescriptions. In recent years, several studies on RTX use in the pediatric population have emerged, which will hopefully lead to a more structured and evidence-based approach to the management of these patients. Long-term, prospective, multicenter studies are especially important to develop consensus recommendations on the ideal dosage and duration of RTX therapy in children, taking into consideration both the potential risk of long-term toxicity and the role of concomitant immunosuppression.
